# Regulating Availability: How Access to Alcohol Affects Drinking and Problems in Youth and Adults

**Published:** 2011

**Authors:** Paul J. Gruenewald

**Keywords:** Alcohol consumption, problematic alcohol use, public policy on alcohol and other drugs, community-based prevention, public policy on alcohol, prevention through decreasing availability and accessibility, alcoholic beverage sales outlet, alcohol availability, alcoholic beverage distribution laws, hours and days of alcohol sales, alcoholic beverage control system, ABC monopoly system, minimum drinking age laws, underage drinking

## Abstract

Regulations on the availability of alcohol have been used to moderate alcohol problems in communities throughout the world for thousands of years. In the latter half of the 20th century, quantitative studies of the effects of these regulations on drinking and related problems began in earnest as public health practitioners began to recognize the full extent of the harmful consequences related to drinking. This article briefly outlines the history of this work over four areas, focusing on the minimum legal drinking age, the privatization of alcohol control systems, outlet densities, and hours and days of sale. Some historical background is provided to emphasize the theoretical and empirical roots of this work and to highlight the substantial progress that has been made in each area. In general, this assessment suggests that higher minimum legal drinking ages, greater monopoly controls over alcohol sales, lower outlet numbers and reduced outlet densities, and limited hours and days of sale can effectively reduce alcohol sales, use, and problems. There are, however, substantial gaps in the research literature and a near absence of the quantitative theoretical work needed to direct alcohol-control efforts. Local community responses to alcohol policies are complex and heterogeneous, sometimes reinforcing and sometimes mitigating the effects of availability regulations. Quantitative models of policy effects are essential to accelerate progress toward the formulation and testing of optimal control strategies for the reduction of alcohol problems.

In addition to increasing beverage taxes or prices as a means to reduce alcohol accessibility, policymakers also may limit alcohol availability through laws and regulations that (1) proscribe sales to underage youth; (2) allow the monopolization of production, distribution, or sales of alcohol; and (3) reduce physical access to alcohol by reducing numbers of outlets or limiting hours and days of sale. These restrictions on availability have been declared effective for reducing alcohol abuse and related problems in major policy reviews (Anderson et al. 2009; Campbell et al. 2009; Popova et al. 2009) and often are the focus of community-based prevention programs (e.g., Guide to Community Preventive Services 2011). This article provides a brief history of availability studies over the past 60 years, points out the limitations of some of this work, and provides some guidelines for the future. It concludes with some general observations about the social ecology of alcohol outlets and suggests how this larger conceptual framework can integrate these research efforts. The intent is to provide a guide to relevant concepts, literature, and research questions for students and researchers new to this area of study.

## Historical Background

With the exception of the United States’ experiment with prohibition, policymakers generally have taken more moderate approaches to regulating the availability of alcohol. In the early and mid-20th century, policymakers in Scandinavia and the United Kingdom experimented with regulations intended to reduce or minimize alcohol problems by rationing alcohol, monopolizing sales through State agencies, and otherwise restricting alcohol markets. Alcohol-policy researchers benefited from these experiments when science-based alcohol-policy studies were pioneered by researchers in these countries (see Babor et al. 2003; Bruun et al. 1975; Edwards et al. 1994). Alcohol-policy research in the United States lagged far behind, with the earliest work performed by economists in the 1960s (e.g., Simon 1966) and modern quantitative studies getting under way in the 1980s. Hoadley and colleagues (1984) and Ornstein and Hannsens (1985) performed the first large-scale, State-level statistical analyses of alcohol-control laws and their relationships to alcohol sales, suggesting that populations living in monopoly States, or States with other restrictive control systems, drank less and had fewer alcohol-related problems.

Theoretical approaches to understanding the effects of alcohol-control regulations on alcohol use and problems were in a fairly primitive state in the 1980s. Researchers relied upon some general assumptions about the effects of regulation on the costs of alcohol (e.g., convenience costs plus real costs summarized as full costs; see Chaloupka et al. 1998) and the Ledermann hypothesis (Ledermann 1956), which posits that changes in average drinking levels would affect heavy use and problems. The full-cost model assumed that reduced availability would increase the costs of alcohol to individual drinkers, resulting in decreased purchases, use, and problems. The Ledermann hypothesis restated observed statistical associations between average use and problems, occasionally rationalized by reference to general forms of social or cultural influence (Gmel and Rehm 2000; Skog 1985). Both approaches received general support in the research literature (see Single 1988), but neither adequately addressed the structural aspects of alcohol-distribution systems (e.g., the effects of alcohol monopolies; see Cook 2007) or the specific effects of drinking contexts on problems (e.g., violent assaults related to alcohol outlets; see Parker 1993). More comprehensive approaches to detailing the social ecological mechanisms that shape drinking patterns and behaviors began to appear in the 1990s (Babor et al. 2003; Parker 1993; Stockwell and Gruenewald 2001). Before discussing this theoretical work, the following section will review the many empirical advances that were made during this time.

## Availability Regulation

States regulate many aspects of alcohol availability, from the age at which someone can purchase alcohol to the types of stores where alcohol is sold and the location and hours of operation of those stores. Research has examined each of these aspects, as outlined below.

## Minimum Legal Drinking Age

Alcohol regulation in the United States is exemplified by the minimum legal drinking age (MLDA). Until 1984, individual States had established different minimum ages at which alcohol could be purchased from retail outlets. Among States that allowed alcohol sales, some established the MLDA at age 18, others at age 21, some at age 18 for beer and age 21 for liquor, and so forth. In a landmark series of studies, Wagenaar and colleagues (see O’Malley and Wagenaar 1991; Wagenaar 1993; Wagenaar and Wolfson 1995) demonstrated that when States switched to a higher (or lower) MLDA, use and problems decreased (or increased) among underage drinkers. This pattern of effects continued until 1984, when all States were encouraged to adopt an MLDA of 21. Higher MLDAs make it more difficult for underage drinkers to purchase alcohol, reduce drinking among underage youth, reduce drinking among of-age youth who grow up with higher MLDAs, and reduce alcohol-related motor-vehicle crashes and other problems (Wagenaar and Toomey 2002). MLDA laws are effective, relatively easy to implement and enforce, and, although underage youth still can obtain alcohol through other means, generally are beneficial to society, saving the lives of up to 1,000 young people each year (Shults et al. 2001; Wagenaar and Wolfson 1995; Wechsler and Nelson 2010;).

Because MLDAs in the United States have not changed for a number of decades, current research looks back in time to reexamine fatal-crash rates among underage drinkers, exploring contingencies in policy effectiveness related to enforcement and support for MLDA laws (Miron and Teitlebaum 2009) as well as other constraints on the alcohol market (e.g., taxes) (Ponicki et al. 2007). This work has been reinforced by efforts to specify alcohol involvement in these crashes using blood alcohol content imputation techniques, which indicate very substantial reductions related to underage alcohol use (Fell et al. 2009). Recent innovative work also has examined the long-term effects of MLDAs on past-year alcohol and drug use disorders among of-age and aging adults, which demonstrates the long-term beneficial effects of these laws on adult drinking behaviors (Norberg et al. 2009).

Despite this evidence, some countries still have low MLDAs, such as Germany at age 16, and other countries have lowered the MLDA in recent years. New Zealand lowered its minimum purchase age from 20 to 18 in 1999, apparently causing increases in hospital emergency-department admissions for intoxication (Everitt and Jones 2002), prosecutions for drunken driving (Guria et al. 2003), and fatal and nonfatal traffic crashes (Huckle et al. 2002; Kypri et al. 2006). This was accompanied by modest increases in use among newly of-age youth aged 18 and 19 and, as a matter of some concern, larger increases among underage youth aged 16 and 17 (Huckle et al. 2010), with indications that older youth were providing alcohol to underage drinkers (Huckle et al. 2008).

On the basis of these observations, one would question why any government would lower the MLDA below age 21. The many answers to this question include the willingness of governments to neglect public health for commercial interests and limits to the science base supporting MLDA policies. Governments may argue that expanded tax receipts from commercial operations will be beneficial to the public without weighing these benefits against the costs associated with drinking (Cook 2007). Advocates of lowered MLDAs may argue that young people can learn to drink moderately in safe drinking environments, such as publicly regulated bars and taverns, without specific evidence that drinking in these contexts is associated with lower drinking risks (as suggested by the Amethyst Initiative 2010). Such arguments can be supported or refuted by sustained research efforts in these important areas.

## Privatization and the Elimination of State Alcohol Controls: Deconstructing Alcohol Monopolies

After the end of prohibition in the United States in 1933, States were allowed to establish either “monopoly” or “license” systems to regulate alcohol production, distribution, and sales. Monopoly systems monopolized some aspects of the alcohol trade. License systems licensed production, distribution, and sales through commercial establishments. No pure monopoly system was established in any State, but partial monopolies were established, most often monopolizing retail sales of one beverage (usually liquor) or another. As a general rule, monopoly States also had more restrictions on licensed aspects of the alcohol trade, whereas license States had more liberal policies. Because States could choose, and often did choose, to regulate alcohol sales in uniquely different ways in response to different public and commercial pressures, a hodgepodge of alcohol regulation resulted that remains a policy night-mare to this day (see Gruenewald and Janes 1991; Gruenewald et al. 1992). Therefore, researchers must examine each State’s regulatory apparatus separately.

After 1933, U.S. alcohol policy was characterized by successive waves of deregulation (MLDA laws are an unusual exception in this regard). Beginning in the 1980s, there was a broad movement among States to privatize aspects of alcohol monopolies, reduce government involvement in alcohol sales, and increase State revenues through alcohol taxes. Early work by Smart (1986) and Macdonald (1986) focused on concurrent changes in Canadian and U.S. alcohol-control systems and indicated that relaxed alcohol controls were related to greater sales and problems. This work was given a substantial boost in the United States when Holder and Blose (1987) examined provision of liquor-by-the-drink options at on-premise outlets in North Carolina. They showed that both alcohol use and related motor vehicle crashes increased substantially after North Carolinians were provided the opportunity to purchase liquor by the drink at bars and restaurants. Another landmark series of studies by Holder and colleagues (Holder and Wagenaar 1990; Wagenaar and Holder 1991, 1995) followed, demonstrating similar effects of different privatization steps in five additional States.

The privatization of alcohol sales in the United States has proceeded, like all alcohol regulation, piecemeal and in fits and starts, depending on State regulatory and political environments. Privatization provisions may include allowing wine sales in grocery stores, liquor sales at bars, the elimination of State stores run by alcohol monopolies, allowances for credit card sales, and so on. Comprehensive policy studies of continuing privatization steps in the United States and their effects on alcohol sales and problems are critically needed. Looking again to Canada, recent detailed studies of the privatization of off-premise sales in British Columbia repeat the findings that privatization generally leads to increased sales and problems but with a new twist; the effects depend on the local mix of newly privatized versus State liquor outlets in an area (Stockwell et al. 2009, 2011). The local effects of global privatization efforts can be substantial.

## Retail Availability: Outlet Density

Three aspects of alcohol availability are regulated to some extent by all U.S. States. These include the type, number, and permissible locations of alcohol outlets. In general, on-premise outlets, (i.e., those that permit use at the point of purchase) are regulated somewhat differently than off-premise outlets, (i.e., those that allow take-away sales and do not typically permit use at point of purchase). Historically, on-premise outlets have been the subject of more stringent regulation because they have been perceived as exposing populations to greater health risks, such as heavy use, public drunkenness, drunken driving, and violence. Off-premise outlets also have been related to signs of civil disorder, however, thus stimulating questions about the roles alcohol outlets play in the etiology of community health problems and making such questions a matter of public health interest throughout the world (Hadfield 2009).

The economic geography of alcohol outlets is little studied but important to consider whenever relationships between outlets and problems are explored. Greater demand for alcohol will lead to the opening of greater numbers of outlets, these outlets will cluster where consumer activities are greatest (e.g., entertainment areas), and the numbers and types of outlets will proliferate until demand is met. Greater numbers of outlets will tend to open in areas where rents are low, resulting in higher concentrations in low-income areas (Gorman and Speer 1997) and some additional exposure of these populations to risks associated with these drinking places. Scientific studies of the effects of alcohol outlets on community health lead to fundamental questions about the social ecology of human behaviors. As reviewed below, researchers have sought to determine whether regulating the number, types, and locations of outlets can lead to fewer public health problems and safer communities.

Early international work had indicated that, short of prohibition, regulations on outlet densities could ameliorate community problems, such as public drunkenness and violence (Edwards et al. 1994). U.S. studies from the 1980s suggested that per capita numbers of alcohol outlets were correlated with both chronic and acute outcomes related to alcohol use (Colon et al. 1983; Watts and Rabow 1983). At that point, the literature confronted two problems: first, no measures of alcohol sales were available, so researchers could not distinguish outlet effects per se from those related to actual alcohol sales, and second, it is difficult to distinguish the ecological effects of outlet concentrations from other ecological correlates of problems across community areas.

In response to the first concern, data from Norway found substantive relationships between measures of outlet densities, sales, and violence related to alcohol use (Bye 2007; Norstrom 2000). In the United States, where data on outlet densities are sparsely collected and sales data are only are collected at the State level, statistical assessments of relationships between densities, sales, and use are rare. Although one State-level panel study suggested relationships between some measures of outlet density and alcohol sales (Gruenewald et al. 1993), limitations of available data precluded replication of this work. Survey-based estimates of alcohol use also have been related to numbers of outlets across community areas, but the results have been mixed (Gruenewald et al. 2002; Pollack et al. 2004). It is notable, however, that whenever disaggregated sales data are available (usually from sources outside the United States), the number and density of alcohol outlets is shown to predict sales and problems (Gruenewald et al. 1999; Stevenson et al. 1999; Stockwell et al. 2009).

In response to the second concern, the rapid development of hardware and software architectures for the representation and analysis of geographic data have enabled the rapid development of scientific studies of outlets and problems. Population-based analyses of these relationships became very active in the early 1990s with the work of public health epidemiologists (Scribner et al. 1994, 1995), criminologists (Roncek and Maier 1991), and economists (Jewell and Brown 1995). These analyses suggested significant and substantive relationships between outlet densities, alcohol-related traffic crashes, violence, and crime. Emerging somewhat later, spatial statistical analyses, which identified and corrected for statistical biases that arise in analyses of these spatial data (e.g., spatial autocorrelation) fully validated this early work (Gorman et al. 2001; Gruenewald et al. 1996; Lipton and Gruenewald 2002). Current spatial statistical models allow researchers to distinguish outlet-specific effects from a host of ecological confounders in urban and rural studies (e.g., Britt et al. 2005; Gruenewald et al. 2006; Wood and Gruenewald 2006; Zhu et al. 2006) and to examine data from geographic units over time, providing insights into the longitudinal dynamics of outlets and problems (Banerjee et al. 2008; Gruenewald and Remer 2006; Livingston 2008; Roman et al. 2008).

This research has led to four empirical generalizations: (1) Whenever alcohol sales can be measured, greater outlet densities are directly related to use; (2) greater densities of bars and taverns and similar on-premise drinking places are directly related to assaults and violence; (3) greater densities of bars, taverns, and sometimes restaurants are directly related to drunken driving and alcohol-related crashes; and (4) spatial effects in these analyses are large, require spatial statistical techniques for unbiased analysis, and suggest the presence of unmeasured correlated effects between geographic areas (i.e., the effects of outlet concentrations in one area have an impact on problems in another).

### Public Policy and Social Ecological Theory

Empirical demonstrations that some public health problems are statistically related to alcohol outlets provide little theoretical guidance to the origin of the problems related to those outlets. When policymakers hear that they can reduce community problems by regulating the numbers and densities of outlets, they quite reasonably ask, “Which outlets should be regulated and where?” Some outlets are high risk; others are not. Some areas in which alcohol outlets are located are the source of much crime; others are not. For effective regulation, quantitative theoretical models are needed to provide estimates of the effects of regulatory controls in different environmental contexts. Research is needed to develop adequate social ecological theory to explain environmentally specific outlet effects.

Not surprisingly, criminologists are interested in relationships between outlets and violent crime. Roncek and Maier (1991) suggested that people routinely meet and drink in outlets, exposing them to risk for violence (routine activity theory), and that neighborhoods with many outlets tend to be socially disorganized, predisposing them to violence (social disorganization theory). Parker (1993) proposed that alcohol outlets are crime attractors, providing places where potential criminals can meet and interact and support one another’s problem behaviors. Scribner and colleagues (2008, 2010) suggested that drinkers attracted to outlets form core groups in which drinkers mutually support problem behaviors, like drinking and driving, maintaining health problems in community systems. Combining the theory that outlets attract crime with the idea that they also facilitate the formation of core groups, Gruenewald (2007, 2008) argued that the commercial activities of outlets encourage the formation of problem drinking groups in high-density outlet areas, reinforcing the link between outlets and crime. These theoretical models move beyond the methodological individualism of full-cost theories to incorporate social processes (e.g., network formation and assortative mixing) into explanations of problems related to outlets. Although much of this work is in development, predictions from these models are practical and eminently testable (see Treno et al. 2007).

Interest in both the theoretical and empirical bases for outlet effects has grown enormously over the past 10 years, in large part as a result of the striking and troubling observations that outlet densities seem related to rates of child abuse and neglect (Freisthler et al. 2008), intimate partner violence (Cunradi 2011; Livingston 2011; McKinney et al. 2009), sexually transmitted diseases (Scribner et al. 2008), college drinking (Weitzman et al. 2003), and injuries among youth and young adults (Gruenewald et al. 2009). Over-concentrations of alcohol outlets also may be a source of increased exposure to injury risks among poor and minority groups in the United States (LaVeist and Wallace 2000; Romley et al. 2007). Theoretical explication of the social mechanisms that relate outlets to these problems is crucial to identifying the full effects of regulating outlets in community settings.

## Retail Availability: Hours and Days of Sale

Regulations on outlet density often are supplemented by restrictions on the hours and days that alcohol can be sold. Examples of these restrictions include Sunday “blue laws,” which originally precluded alcohol sales for religious reasons, and regulations on hours of sale common to all States. The effects of these restrictions on alcohol use and problems are widely debated, with advocates claiming positive effects and opponents arguing that, at best, these restrictions serve to redistribute use and problems to other days and times. For example, the United Kingdom’s Licensing Act of 2003 allowed staggered closing hours for outlets under the assumption that common closing hours increased crowding and alcohol-related crime (Humphreys and Eisner 2010; Treno 2010). This may or may not be the case. Regardless, it demonstrates governments’ willingness to change these regulations in the absence of scientific evidence.

Unlike policies whose effects are difficult to avoid, such as changes in alcoholic-beverage taxes and outlet densities, reductions in hours and days of sale may be rendered ineffective if drinkers displace their drinking to other days or times. Whether displacement actually occurs in any given instance is an empirical question that bedeviled empirical research in the 1980s and 1990s. Recent work suggests that displacement may not be a substantive issue and that extended days of sale (in the United States, McMillan and Lapham 2006; in Sweden, Norstrom and Skog 2005) and later trading hours (in Australia, Chikritzhs and Stockwell 2002, 2006) may both be related to increases in drinking and problems. Later trading hours have been particularly associated with increased homicides (in Brazil, Duailabi et al. 2007) and alcohol-involved emergency-department admissions, especially assaults (in London, Newton et al. 2007).

Studying the effects of limiting the days and hours of alcohol sales has been especially difficult in the United States because suitable natural experiments by which to test these effects rarely have occurred. Changes in hours and days of sale typically take place as part of a bundle of other privatization steps (see above), and this makes it very difficult to disentangle policy effects. At best, the international literature suggests that relaxed trading hours for on-premise places like bars and clubs may lead to increases in drinking and problems (Stockwell and Chikritzhs 2009), that increased days of sale also may be related to greater problems; yet the findings in both of these areas remain inconsistent (e.g., Vingilis et al. 2005, 2006). Further clarity with regard to these potential policy effects can be achieved through the development of explicit theoretical models of the effects of hours and days of sale on drinking and problems, supplemented by specific models of displacement. Future empirical work would benefit from the direction provided by such models.

## Regulating Youth Access: Local Regulatory Policy, College Drinkers, and Underage Youth

As a general rule, regulations on availability in developed countries single out one demographic group as specifically subject to restriction: underage youth whose early drinking onset may lead to greater drinking problems later in life (Windle et al. 2009). As noted above, MLDA laws are an effective, although permeable, barrier to alcohol use among underage drinkers. With sufficient motivation, underage drinkers can and do obtain alcoholic beverages. Early research indicated that between 30 and 70 percent of purchase attempts by underage drinkers at off-premise outlets were likely to be successful (Forster et al. 1994) but that consistent enforcement efforts could drive these figures much lower (Grube 1997). These figures and effects mostly are unchanged to this day (Paschall et al. 2010*a*). For this reason, preventing alcohol sales to minors through enforcement efforts is a key feature of community-based alcohol-prevention programs intended to reinforce MLDA effects (e.g., Holder et al. 2000; Wagenaar et al. 2000).

Because the MLDA deterrent is permeable, researchers have become interested in the extent to which outlet densities, hours and days of sale, and other regulations specific to youth may affect underage drinking and problems (Grube 2009). This relatively new area of research is of special interest because alcoholic beverages are starting to be regulated in ways similar to other illegal drugs. States are extending MLDA laws to proscriptions on possession and use (National Institute on Alcohol Abuse and Alcoholism [NIAAA] 2010). Thus, much like the illegal-drug market, underage alcohol use is linked to access through informal familial and social networks (LaScala et al. 2005; Treno et al. 2008). Indeed, the most common sources of alcohol among underage drinkers are through the home and friends (Paschall et al. 2010*b*; Wagenaar et al. 1996). Thus, initial studies suggest that underage purchases are more likely to be successful among outlets that are more densely clustered (Friesthler et al. 2003), that greater outlet densities are related to teenage drinking (Huckle et al. 2008; Jones-Webb et al. 1997; Livingston et al. 2008; Truong and Strum 2009), that access through social networks may mediate or moderate these effects (Chen et al. 2009, 2010), and that outlet densities may be related to drinking and drunken driving among youth (Grube and Stewart 2004; Treno et al. 2003).

## Future Directions

Most policies and regulations that are intended to restrict the availability of alcohol are applied through retail alcohol outlets. Retail outlets set the final prices at which alcohol will be sold to drinkers, restrict sales to of-age patrons (at least to some degree), choose locations at which to open and compete, and determine their own hours and days of sale within the limits set by law. Retail alcohol outlets are the formal social structures through which drinkers obtain alcoholic beverages, whether they use them onsite or carry them away for use elsewhere. These facts may sometimes elude researchers when they focus on estimating the effects of a global measure of policy change on use or problems (e.g., a tax increase). But the primacy of these contexts in the regulation of drinking behaviors remains. Therefore, ecological studies of alcohol outlets are of central importance to the field.

Community systems theorists (Holder 1998) and social ecologists (e.g., Gruenewald 2007; Parker 1993; Scribner et al. 2010) have recognized for some time the key roles that alcohol outlets play in the etiologies of harmful alcohol use and related problems. These theoretical approaches reinforce the importance of empirical work, which focuses on the social mechanisms by which regulations on availability affect the distribution of problems related to alcohol. Simply put, understanding the effects of regulations on drinking in context is the key to understanding the effects of limiting availability. A hypothetical case helps make this point: It is quite possible to imagine conditions in which a higher MLDA could lead to both less use and more problems. A higher MLDA could lead to less drinking among a large number of drinkers in low-risk contexts, decreasing use, and more drinking among a smaller number of drinkers in high-risk contexts, increasing problems. A naïve statistical assessment of overall policy effects would suggest that higher MLDAs should be preferred to prevent use but lower MLDAs should be preferred to prevent problems. A refined study of use in contexts, however, would demonstrate heterogeneous effects related to the local characteristics of drinkers and the availability of high- and low-risk drinking settings. In this way, local contexts can reinforce or mitigate the impacts of global alcohol policy.

Obviously, this thought problem leads to a number of important questions about drinking contexts: What are high- and low-risk contexts? Are these places populated by high- and low-risk drinkers? How do drinkers mix in these contexts? And, at a larger scale, does the number of different contexts in a community contribute to the risks experienced by drinkers? These questions, and others like them, are just beginning to be explored as theoretical models guide research into how drinkers segregate into drinking contexts (Gruenewald 2007), the effects of mixing among drinkers in these contexts (Mubayi et al. 2011), and the global effects of drinking environments on etiology of drinking problems across these contexts (Rosul et al. 2011). Efforts to pose and answer these and related questions will provide foundations for finally understanding the social mechanisms by which alcohol environments affect alcohol problems (Gruenewald et al. 1993). In turn, answers to these questions can guide community prevention efforts. The new contribution of current social ecological models is to begin to provide connecting theory that links global alcohol policies to context-specific risks in community settings. Regulations on availability directly affect the formal operations of commercial establishments, patterns of drinking in those establishments, and associated risks. These regulations also indirectly affect drinking in other contexts where alcohol is used and other risks may arise, such as at parties and social gatherings and in the home. The total effect of any alcohol policy is to change the system of relationships between contexts, use, and problems across communities, with the expectation that there will be some remediation in harms related to use. It is at the junctures of these systems of relationships that the most effective environmental prevention programs can be built.
